# Does micronutrient supplementation improve outcomes in adult gastrointestinal surgery patients? A systematic review

**DOI:** 10.3389/fnut.2025.1719617

**Published:** 2025-12-12

**Authors:** Daoud Salman, Trevor Smith, Philip C. Calder

**Affiliations:** 1 Faculty of Medicine, School of Human Development and Health, University of Southampton, Southampton, United Kingdom; 2 University Hospital Southampton NHS Foundation Trust, Southampton, United Kingdom; 3 NIHR Southampton Biomedical Research Centre, University Hospital Southampton NHS Foundation Trust and University of Southampton, Southampton, United Kingdom

**Keywords:** micronutrients, gastrointestinal surgery, surgical complications, bariatric, vitamins, trace elements, antioxidants, oxidative stress

## Abstract

**Introduction:**

Gastrointestinal surgery can result in short-term (in hospital) complications such as infections, post-operative ileus and poor wound healing and longer-term (post-discharge) complications such as intestinal failure, malnutrition and loss of bone mass. These complications can severely impact the patient and increase healthcare costs. Impaired immunity, excessive inflammation and oxidative stress can contribute to postoperative complications and poor outcome. Many micronutrients support immunity and help to control inflammation and oxidative stress. Therefore, providing micronutrients could mitigate poor outcome from surgery. The aim of this systematic review was to collate findings from randomized controlled trials (RCTs) of micronutrients provided to adult patients undergoing, or who had undergone, gastrointestinal surgery.

**Methods:**

Searches were conducted in Medline and CINHAL; only literature from 2014 onwards was searched.

**Results:**

A total of 12 articles reporting data from 11 RCTs were included. These trials studied vitamin D alone (administered orally) or combinations of different micronutrients (administered orally, intravenously or in wound dressings). Six trials started the intervention following discharge from hospital. Six trials had a low risk of bias overall, while five had some concerns. Two trials found that vitamin D decreased the loss of bone density in those who had undergone bariatric surgery, but two other trials did not find this; vitamin D dose may be important in determining its effect. One trial found that vitamin D improved quality of life in those who had surgery and another found that vitamin D improved survival time and prevented relapse in some patients who had surgery for gastrointestinal cancer. Another trial found that vitamin D helped support the immune response following surgery; this could lead to fewer infections, although that outcome was not reported. Intravenous multivitamins shortened hospital stay and decreased oxidative stress in the immediate postoperative period in one trial. Using vitamin E and silicone in wound dressings decreased surgical site infection, postoperative pain, inflammation and hospital stay in one trial.

**Discussion:**

Although some micronutrients may reduce risk of short- and long-term complications of surgery, insufficient trials have been conducted to make strong conclusions. Further research, especially with interventions in the perioperative period and looking at in-hospital outcomes, is needed.

## Introduction

Patients undergoing gastrointestinal surgery may develop different short- or long-term complications. Short-term (in-hospital) complications include infections, post-operative ileus and poor wound healing which can lead to prolonged hospital stay (1). Impaired immune function and heightened oxidative stress and inflammation can play a central role in increasing the risk of these short-term complications (2–6). Long-term complications (i.e., occurring some time—days, weeks, months or even years—post discharge) include intestinal failure, malnutrition and loss of bone mass, especially in those who have undergone bariatric surgery, resulting in increased fracture risk (7). Both short- and long-term surgical complications are associated with decreased quality of life, increased morbidity and mortality and increased healthcare costs (8–10).

Micronutrients are vitamins and trace elements that are essential to human metabolism and physiology (11). Low intakes of certain micronutrients, resulting in low status (i.e., low blood concentrations) are common in the general population (12). Furthermore, patients undergoing gastrointestinal surgery may have sub-optimal micronutrient status due to poor diet or due the presence of disease adversely impacting food intake, digestion and absorption (13). Following gastrointestinal surgery patients may experience reduced food intake (this is long-term in those who have undergone bariatric surgery) and this can result in low intake and status of micronutrients (14). There may be a link between low micronutrient intake and status and post-surgical complications. This is because of the role of multiple micronutrients in supporting immune function and wound healing and in controlling oxidative stress and inflammation (11, 15–18). Micronutrients that support immunity include vitamin A (19, 20), the B vitamins (21, 22), vitamin C (23, 24), vitamin D (25, 26), zinc (27, 28), copper (29, 30), iron (31, 32) and selenium (33, 34). Vitamin C is also especially important to wound healing (35). Antioxidant micronutrients act to decrease oxidative stress or mitigate its effects and include vitamins C and E and the trace elements that form part of the active site of the protective enzymes superoxide dismutase (manganese, copper, zinc), catalase (iron) and glutathione peroxidase (selenium). Oxidative stress promotes inflammation (36, 37) meaning that antioxidant micronutrients are also anti-inflammatory. Micronutrients can also be anti-inflammatory through their inhibitory effects on the production of pro-inflammatory lipid and protein mediators of inflammation (11). Thus, low intakes and status of a number of micronutrients can impair immunity and wound healing and promote oxidative stress and inflammation, placing surgical patients at risk of poor outcome in the immediate post-operative period. There may also be longer-term impacts of low micronutrient intake or uptake on surgical patients. For example, the long-term effects of bariatric surgery on bone mass and fracture risk may relate to decreased absorption of vitamin D, especially since this occurs primarily in the jejunum and ileum which are both commonly excluded in bariatric surgery (38). Additionally, the vitamin D deficiency that can result from bariatric surgery can lead to secondary hyperparathyroidism which is associated with osteoporosis and fractures (39). Even the risk of developing pathologies such as Parkinson Disease may be increased after bariatric surgery, where there is associated deficiency of vitamin B12 (40). Therefore, provision of micronutrients may be useful in patients undergoing, or who have undergone, gastrointestinal surgery and they may reduce the risk of both short-term and long-term complications.

Supporting the notion that micronutrient provision might be effective in reducing post-surgical complications are the observations that higher post-operative vitamin D levels are associated with improved survival in patients with colorectal cancer who underwent surgery (41) and that vitamin D deficiency was associated with increased pain and opioid use following colorectal cancer surgery (42). Despite these observations, there is yet to be an analysis of the existing randomized controlled trials (RCTs) involving the use of perioperative micronutrient supplementation. Therefore, the aim of this systematic review is to determine if micronutrient supplementation in the perioperative period can reduce the incidence of complications in adult patients undergoing gastrointestinal surgery. Anticipated short-term (in-hospital) complications included infection, impaired wound healing, oxidative stress, inflammation, impaired immune response, prolonged hospital stay and death while anticipated long-term (post-discharge) complications included disease recurrence, reduced quality of life, reduced bone health, and death.

## Materials and methods

### Overview

The PICO (Patient or Population, Intervention, Comparison and Outcome) approach was used to produce key terms. The population was patients aged 18 years or over (i.e., adult males and females) undergoing gastrointestinal surgery. The intervention was perioperative micronutrient supplementation (single or combination of micronutrients before, during or after surgery). The comparison was between patients who had received micronutrient supplementation and those who had not. The outcome was the presence of short- or long-term surgical complications (e.g., infection, impaired wound healing, prolonged hospital stay and death as short-term in-hospital complications and disease recurrence, reduced quality of life, reduced bone health and death as long-term post-discharge complications) or related biomarkers (e.g., markers of immune function, inflammation or oxidative stress). The systematic review was conducted according to the “Preferred Reporting Items for Systematic review and Meta-Analysis” (PRISMA) (43) guidelines and was done for educational purposes so therefore was not registered.

### Literature search

Because of restrictions on time, the literature searching was restricted to two databases, OVID MEDLINE and CINHAL. These databases were searched for articles published from 1st January 2014 in order that findings would be relevant to current clinical practice. Literature searches were performed in October 2024. Search terms were: (“micronutrient*,” “micro nutrient*,” “trace element*,” “antioxidant*,” “mineral*,” “vitamin*”) combined with (“perioperative,” “peri operative,” “surgery,” “surgical,” “preoperative,” “pre-operative,” “postoperative,” “post-operative”) combined with (“gastrointestinal,” “gastric,” “bowel,” “rectum,” “rectal,” “intestinal” “colorectal” and “bariatric”).

### Article selection

Once the articles were identified by the searches, they were selected for inclusion based on the following criteria: describing a randomized control trial; involved the use of micronutrient supplementation (single or combination of micronutrients) pre or post-surgery as an intervention; involved gastrointestinal surgery (which includes bariatric and colorectal surgery); included patients were all aged 18 years or older; article was fully accessible; article was published in the English language; reported on one or more surgical complications. Anticipated short-term complications included infection, impaired wound healing, oxidative stress (defined by an elevated blood concentration of a recognized biomarker of oxidative stress such as malondialdehyde), inflammation (defined by an elevated blood concentration of a recognized biomarker of inflammation such as C-reactive protein, interleukin-6 or total leukocytes), impaired immune response (defined by an altered number or ratio of blood immune cells or a lower functional response of an immune cell population tested ex vivo), prolonged hospital stay and death while anticipated long-term complications included disease recurrence, reduced quality of life, reduced bone health (defined as bone mass or bone mineral density) and death. Exclusion criteria included pancreatic and liver surgeries and any non-gastrointestinal surgeries. Article selection was performed by two authors (DS and PCC); any disagreements were resolved by discussion with the third author (TS).

### Data extraction

Data were extracted from each article by DS and included patient characteristics (clinical state, age and sex), sample size, type of surgery, micronutrient treatment used (including route, dosage and duration), outcomes assessed and when they were measured as well as the conclusions drawn from them.

### Risk of bias assessment

Each trial was assessed for bias using the Cochrane Risk of Bias 2 tool (44). This ranks each study as “low risk,” “some concerns” or “high risk” based on five criteria (domains). The first two domains relate to aspects of trial conduct (the randomization process and deviations from the intended interventions). The other three domains relate to outcomes (missing outcome data, measurement of the outcome, selection of the reported result); these three domains were assessed taking into account all relevant outcome data reported. Finally, an overall risk of bias was made. The findings of were summarized using a traffic light plot.

## Results

### Search results

**Figure 1:**
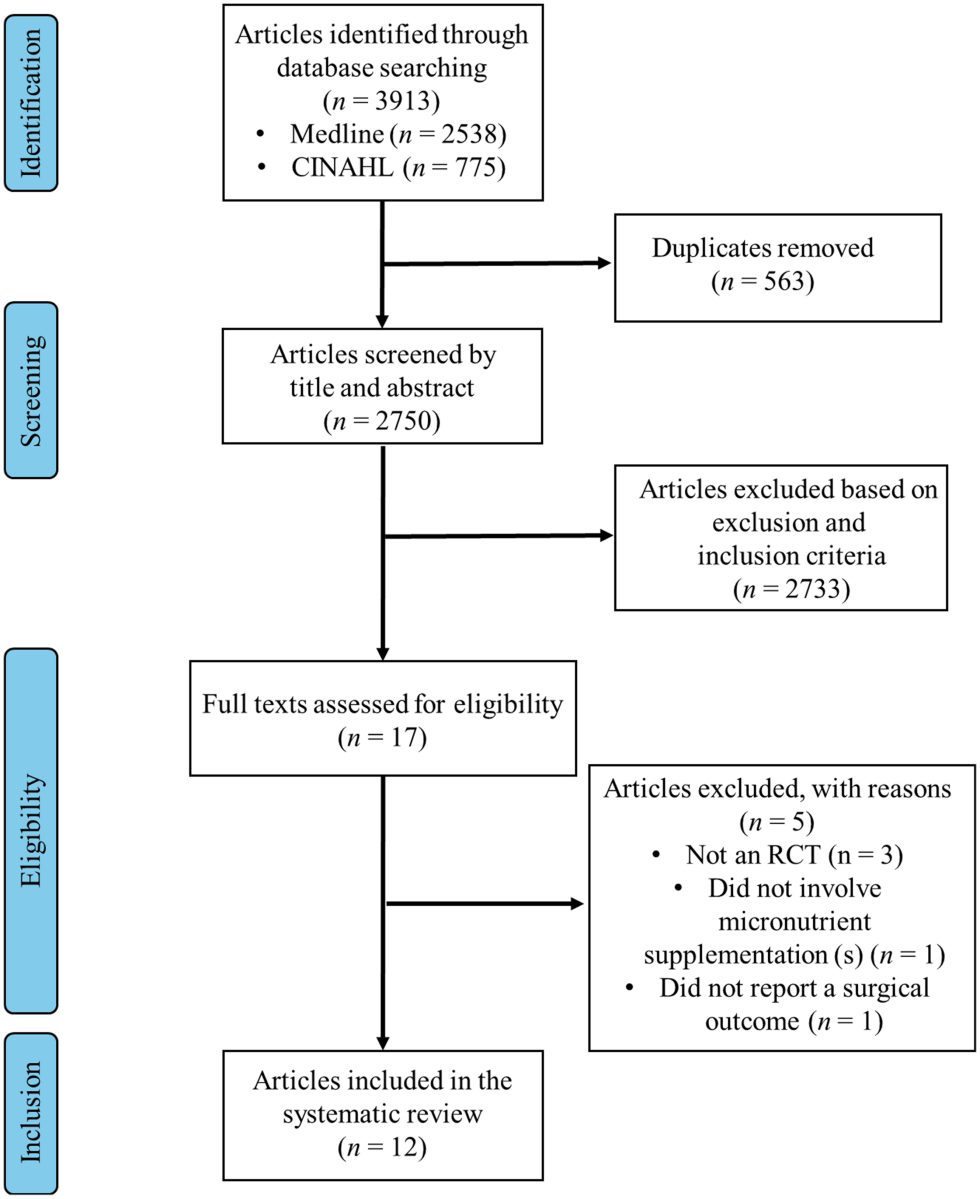
Flow diagram summarizing the identification and selection of articles for inclusion in the systematic review. RCT, randomized controlled trial.

The searches across the two databases returned a total of 3,313 articles (Figure 1); 563 were removed as they were duplicates leaving 2,750 unique articles. These were each screened by title and abstract for relevancy, leaving 17 articles. The full texts of these 17 articles were retrieved. One was removed for not measuring the effect of micronutrient supplementation, one for not measuring surgical complications and three for not being RCTs. This left 12 articles to include in the systematic review (45–56). These 12 articles included data from 11 RCTs; two articles reported data from the same trial (54, 55).

### Trial characteristics

The characteristics of each trial are summarized in Table 1. Of the 11 trials (published in 12 articles) seven (published in 8 articles) used vitamin D (46, 47, 49, 50, 52–55), one used vitamin D and multivitamins (45), one used multivitamins (48), one used multiple micronutrients (56) and one used a combination of vitamin E and silicone (51). Sample size varied from 28 to 1,121 (see Table 1 for details for each trial), with an average of 209. Six trials had a sample size <100 (45, 48, 49, 52, 53, 56). Eight trials administered the intervention orally (45–47, 49, 52–56), one sublingually (50), one intravenously (48) and one embedded in wound dressings (51). Five trials were conducted in patients undergoing different bariatric surgery procedures to treat morbid obesity (45, 49, 50, 53, 56), five (described in six articles) in patients undergoing surgery for some form of gastrointestinal cancer (46, 48, 51, 52, 54, 55), and one in patients undergoing surgery to treat Crohn’s Disease (47). Three trials that used preoperative intervention (45, 50, 56) extended this for some months post-surgery (see Table 1 for details of the duration for each trial). The trial with wound dressings was during the surgery itself (51), while the trial with intravenous administration was during the immediate postoperative period, up to day 6 (48). Two trials started the intervention within 1 month after surgery (47, 53), while the other four trials (reported in 5 articles) started the intervention at least 1 month after surgery (46, 49, 52, 54, 55). Very few of the included trials reported on short-term clinical outcomes; wound healing, anastomotic fistula, hospital stay, and medical costs were reported in the trial of intravenous multivitamin infusion (48). That trial also reported blood inflammatory cytokines and oxidative stress markers at postoperative day 6 (48). The trial of vitamin E and silicone embedded dressings reported hospital stay and post-surgical pain, surgical site infection and blood inflammatory markers 48 h after surgery (51). All other trials had a long follow-up often several years (see Table 1 for details of follow-up for each trial). Consequently, most trials reported on long-term post-discharge outcomes, most commonly related to bone health (45, 49, 50, 53) but also to quality of life (50), disease (colorectal cancer, Crohn’s Disease) recurrence (46, 47), survival (54, 55), or inflammation (47). One trial reported only on blood immune markers (52) and one only on blood markers of inflammation and iron metabolism (56).

**Table 1:** Summary of the characteristics of the included trials.

References	Country	Patient characteristics [clinical state, sex, age (years)]	Number of patients studied (and per group)	Type of surgery	Micronutrient treatment including route, dose and duration	Control	Outcomes of relevance reported and when assessed
Ben-Porat et al. (45)	Israel	Obesity, Female, 18–65	62 (31 control, 31 vitamin D)	Sleeve gastrectomy	Oral multivitamin with vitamin D (4,000 IU /day), 2 months preoperatively	Oral vitamin D (1,200 IU/day)	Bone mineral density at hip, left forearm, lumbar spine and femoral neck; Body weight 2 months preoperatively and 3, 6, 12 months postoperatively
Calderwood et al. (46)	USA	At least one colorectal adenoma removed in past 4 months, male and female, 45–75	1,121 (210 placebo, 215 calcium, 213 vitamin D, 224 vitamin D + calcium)	Endoscopic colorectal adenoma removal	Oral vitamin D (1,000 IU /day), or oral calcium carbonate (350 μg/day) or both starting at least 4 months after surgery until follow up	Placebo	Colorectal adenoma recurrence Either 3 or 5 years after qualifying colonoscopy
De Bruyn et al. (47)	The Netherlands	Crohn’s disease, Male and female, >18	143 (71 placebo, 72 vitamin D)	Ileocecal or ileocolonic resection with ileocolonic anastomosis	Oral vitamin D (25,000 IU weekly) starting within 2 weeks after surgery for 26 weeks	Placebo	Crohn’s recurrence; C-reactive protein; faecal calprotectin; endoscopy At baseline and at weeks 2, 6, 12, and 26
Li et al. (48)	China	Primary gastric adenocarcinoma, male and female, >18	32 (17 placebo, 15 multivitamin)	Radical gastric resection	Parenteral, Total nutrient admixture and multivitamins in the immediate post-operative period	Parenteral, Total nutrient admixture and saline	Wound healing; Anastomotic fistula; Hospital stay; Medical costs; Blood inflammatory cytokines; Oxidative stress (levels of polar metabolites in blood) Pre-surgery and a postoperative day 6
Luger et al. (49)	Austria	Obesity, male, and female, >18	50 (25 placebo, 25 vitamin D)	One-anastomosis gastric bypass	Oral loading doses of vitamin D (3 × 100,000 IU) in first month postoperatively and oral maintenance dose (3,420 IU/day) for next 5 months Then recommended to continue supplementation for next 6 months	Placebo in first month and oral vitamin D (3,420 IU/day) for next 5 months Then recommended to continue supplementation for next 6 months	Bone mineral density at lumbar spine, left hip total body and forearm; Body fat and lean body mass Before then at 6, and 12 months after surgery
Muschitz et al. (50)	Austria	Obesity, male and premenopausal female, > 25	220 (110 control, 110 vitamin D)	Bariatric surgery	Sublingual drops of vitamin D (28,000 IU cholecalciferol per week) for 8 weeks before surgery; then sublingual drops (16,000 IU per week) for 2 years postoperatively Oral calcium monocitrate (1,000 mg/week) Additional exercise and protein supplementation was advised	No treatment	Bone mineral density at lumbar spine, total hip and total body; Bone turnover marker (BTM) via blood test; Body weight, BMI and body composition; Quality of life (via questionnaire) Before surgery and 6, 12, 18, and 24 months post-surgery
Ruiz-Tovar et al. (51)	Spain	Colorectal neoplasia, male and female, mean 67	120 (60 control, 60 vitamin E + silicone)	Elective colorectal laparoscopic surgery	Vitamin E and silicone dressings used for wounds during surgery	Conventional wound dressing	Hospital stay; Pain at 48 h post-surgery; Infection of surgical site; Inflammation (white blood cells and C-reactive protein 48 h post-surgery)
Srichomchey et al. (52)	Thailand	Early-stage colorectal cancer, NR, 30–75	28 (11 control, 17 vitamin D)	Curative surgery (unspecified)	Oral Vitamin D (8,000 IU/day) for 3 months starting at least 1 month after surgery	No treatment	Immune response (% regulatory T cells and cytokines) 1 and 3 months post-surgery
Volonakis et al. (53)	Greece	Obesity, premenopausal female, mean 32	72 (35 placebo, 37 vitamin D)	Long limb-biliopancreatic diversion	Oral Vitamin D (600 IU/day) and calcium (100 mg/day) for 1 year postoperatively; Plus extra vitamin D (10,000 IU/day) during first postoperative month	Oral Vitamin D (600 IU/day) and calcium (100 mg/day) for 1 year	Bone mineral density of lumbar spine Preoperatively and 1 year postoperatively
Urashima et al. (54) and Yonaga et al. (55)	Japan	Digestive tract cancers stages I to III, male and female, 30–90	417 (166 placebo, 251 vitamin D)	Curative surgery	Oral Vitamin D (2,000 IU/day) for 7 years starting 2–4 months post-surgery	Placebo	Relapse-free survival time; Overall survival time; Incidence of other serious events requiring admission; Metastases. Every month for the first 6 months, every 2 months for the second 6 months, every 3 months until 5 years, every 3–6 months after this
Marin et al. (56)	Brazil	Obesity, female, 20–45	45 (11 placebo, 34 micronutrients)	Roux-en-Y gastric bypass surgery	Micronutrient supplementation 30 days preoperatively at the Recommended Dietary Allowance (RDA) and then at twice the RDAs for 6 months postoperatively	Same micronutrient only given 6 months postoperatively	Inflammation (C-reactive protein and cytokines) and iron metabolism (Hematocrit hemoglobin, serum iron and total iron binding capacities) Study entry, after 30 days of micronutrient supplementation during the preoperative period, and at 6 months postoperatively

NR, not reported.

### Trial findings

The trial findings are summarized in Table 2. Of the 11 trials included in this systematic review, eight involved the use of vitamin D supplementation (45–47, 49, 50, 52–55). One provided vitamin D and multivitamins preoperatively (45), one provided vitamin D perioperatively and then post-discharge (50) and the other six (reported in seven publications) provided vitamin D post-discharge (46, 47, 49, 52–55). Most trials looked at long-term outcomes particularly bone mineral density (BMD). Ben-Porat et al. (45) found that preoperative vitamin D along with a multivitamin supplement, had no effect on the decrease in BMD and body weight observed after sleeve gastrectomy. However, Muschitz et al. (50) found the use of vitamin D and calcium perioperatively and post-discharge resulted in smaller losses of BMD (values for vitamin D group are presented before values for the control group: lumbar spine: −1.2% vs. − 7.9% (*p* < 0.001); total hip: −3.9% vs. − 9.9% (*p* < 0.001); total body: −2.0% vs. − 4.1% (*p* < 0.001)) and lean body mass (−3.5% vs. − 12.4% (*p* < 0.001)) than in the control group. There were also lower blood concentrations of bone turnover markers and an overall improvement in quality of life judged by social, emotional, physical and mental factors in the vitamin D group (31). Volonakis et al. (53) found no change in loss of BMD with the post-discharge use of vitamin D in long limb-biliopancreatic diversion patients. In contrast, Luger et al. (49) found that post-discharge vitamin D result in a smaller decrease in BMD in patients who had undergone bariatric surgery than seen in the control group, especially after 12 months (values for vitamin D group are presented before values for the control group: lumbar spine: −5.7 vs. − 10.0%; left hip: −10.0 vs. − 17.4%; forearm: −0.5 vs. − 1.5%; total body: −0.6 vs. − 2.3%). Looking beyond bone health, Calderwood et al. (46) found that post-discharge vitamin D supplementation, along with calcium, did not affect colorectal adenoma recurrence after endoscopic surgical removal. de Bruyn et al. (47) found no association between post-discharge vitamin D supplementation and Crohn’s recurrence after ileocolonic resection. Urashima et al. (54) found that post-discharge vitamin D had no effect on survival time or relapse-free survival time in digestive tract cancer patients. However, when these patients were grouped by histopathological characteristics in a post-hoc analysis (55) these outcomes were improved exclusively in patients with poorly differentiated adenocarcinomas. Srichomchey et al. (52) found post-discharge vitamin D supplements may help support the immune response as they found more Treg cells and higher blood IL-10 (Treg associated cytokine) than in patients in the control group.

**Table 2:** Summary of trial findings.

Reference	Outcomes of relevance reported and when assessed	Effect of micronutrient	Conclusion/interpretation
Ben-Porat et al. (45)	BMD at hip, left forearm, lumbar spine and femoral neck; Body weight To 12 months post-surgery	Multivitamins and vitamin D had no effect on the decrease in BMD and body weight	Preoperative multivitamin and vitamin D supplementation does not affect BMD or body weight following bariatric surgery
Calderwood et al. (46)	Colorectal adenoma recurrence To 5 years post-surgery	Vitamin D and calcium had no effect on adenoma recurrence	Post-discharge vitamin D and calcium supplementation does not affect colorectal adenoma recurrence post-surgery
De Bruyn et al. (47)	Crohn’s recurrence; C-reactive protein and faecal calprotectin; Endoscopy To ~26 weeks post-surgery	Vitamin D had no effect on Crohn’s recurrence or inflammatory markers	Post-discharge vitamin D supplementation does not affect Crohn’s recurrence post ileocecal or ileocolonic resection
Li et al. (48)	Wound healing, anastomotic fistula, hospital stay, economic costs During hospital stay Blood markers of inflammation and oxidative stress At postoperative day 6	Multivitamin supplementation shortened hospital stay and decreased blood oxidative stress markers but did not affect blood markers of inflammation	Intravenous multivitamins in the immediate postoperative period may decrease hospital stay and oxidative stress following bariatric surgery
Luger et al. (49)	BMD at lumbar spine, left hip, total body and forearm; Weight, body fat and lean body mass To 12 months post-surgery	Vitamin D resulted in a smaller loss of BMD	Post-discharge vitamin D supplementation may reduce bone loss in patients following bariatric surgery
Muschitz et al. (50)	BMD at lumbar spine, total hip and total body; Bone turnover marker (BTM) via blood test; Body weight, BMI and lean body mass; Quality of life To 24 months post-surgery	Vitamin D and calcium resulted in smaller loss of BMD and decreased concentration of BTM. Vitamin D and calcium improved social, emotional, physical and mental factors.	Perioperative vitamin D and calcium may reduce bone loss and improve quality of life following bariatric surgery
Ruiz-Tovar et al. (51)	Hospital stay Postoperative pain; Surgical site infection; Blood markers of inflammation At 48 h post-surgery	Vitamin E and silicone in wound dressings decreased postoperative pain, hospital stay and surgical site infections When vitamin E and silicone were used only *Bacteroides fragilis* grew on surgical site while wounds in control group where polymicrobial. Vitamin E and silicone decreased white blood cells and CRP 48 h post-surgery	Vitamin E and silicone in wound dressings may reduce postoperative pain, surgical site infection, inflammation and hospital stay following elective colorectal laparoscopic surgery
Srichomchey et al. (52)	Maintenance of immune response –Tregs in blood and levels of Treg associated cytokines To 3 months post-surgery	Vitamin D increased levels of Tregs and serum IL-10	Post-discharge vitamin D supplementation may support the immune response post-surgery in colorectal cancer patients
Volonakis et al. (53)	BMD of lumbar spine To 1 year post-surgery	Vitamin D had no effect on the decrease of BMD	Post-discharge vitamin D supplementation does not affect bone loss in long limb-biliopancreatic diversion patients
Urashima et al. (54)	Relapse-free survival; Overall Survival time; Incidence of other serious events requiring admission; Metastases To > 5 years post-surgery	Vitamin D had no effect on relapse-free survival or survival time	Post-discharge vitamin D supplementation does not prevent digestive tract cancer relapse
Yonaga et al. (55)	Relapse-free survival time; Overall Survival—according to histopathological characteristics To >5 years post-surgery	Vitamin D improved relapse-free survival time and overall survival in a subgroup of patients with poorly differentiated adenocarcinoma but not in any other group	Post-discharge vitamin D supplementation may reduce relapse and improve survival in patients with poorly differentiated adenocarcinomas of the digestive tract
Marin et al. (56)	Postoperative inflammation and iron metabolism To 6 months post-surgery	Preoperative and postoperative micronutrient supplementation did not affect systemic inflammation but improved iron metabolism	Perioperative micronutrient supplementation does not reduce systemic inflammation but may improve iron metabolism after bariatric surgery

It is important to note that the trial of Ben-Porat et al. (45) described above used preoperative supplementation with vitamin D and a multivitamin. Li et al. (48) found that providing additional vitamin A, B1, B2, B3, B5, B6, B12, C, D, and E and folic acid and biotin as part of parenteral nutrition in the immediate post-operative period resulted in shorter hospital stay (7.1 ± 2.7 vs. 9.3 ± 7.3 days (*p* < 0.05)) and lower blood concentrations of oxidative stress markers following radical gastric resection than seen in the control group. Marin et al. (56), on the other hand found that perioperative oral supplementation with micronutrients (vitamins A, B1, B2, B6, B12, C, D, E and K, nicotinamide, pantothenic acid, folic acid, biotin, magnesium, calcium, zinc, copper, chromium, iron, selenium, manganese, iodine, silicon and vanadium) did not reduce markers of systemic inflammation in women undergoing Roux-en-Y gastric bypass surgery but did improve iron metabolism.

Including vitamin E and silicone in wound dressings used during elective colorectal laparoscopic surgery reduced postoperative pain (values for vitamin E + silicone group are presented before values for the control group: 27.1 ± 10.7 vs. 41.6 ± 16.9 mm on a visual analogue scale; mean difference between control and vitamin E + silicone group 20.5 mm (95% confidence interval 8.4–42.1) *p* < 0.001), surgical site infection (3.4% vs. 17.2% of patients; odds ratio of infection in the control vs. vitamin E + silicone group 6.1 (95% confidence interval 1.27–21.3) *p* = 0.013), hospital stay [median 5 (range 4–26) vs. 7 (range 5–35) days (*p* < 0.001)] and blood markers of inflammation 48 h post-surgery (white cell counts *p* = 0.001; C-reactive protein *p* = 0.016) (51). Additionally, only *Bacteroides fragillis* grew on surgical sites where vitamin E and silicone was used whereas when conventional wound dressings were used wounds were polymicrobial.

### Risk of bias

**Figure 2:**
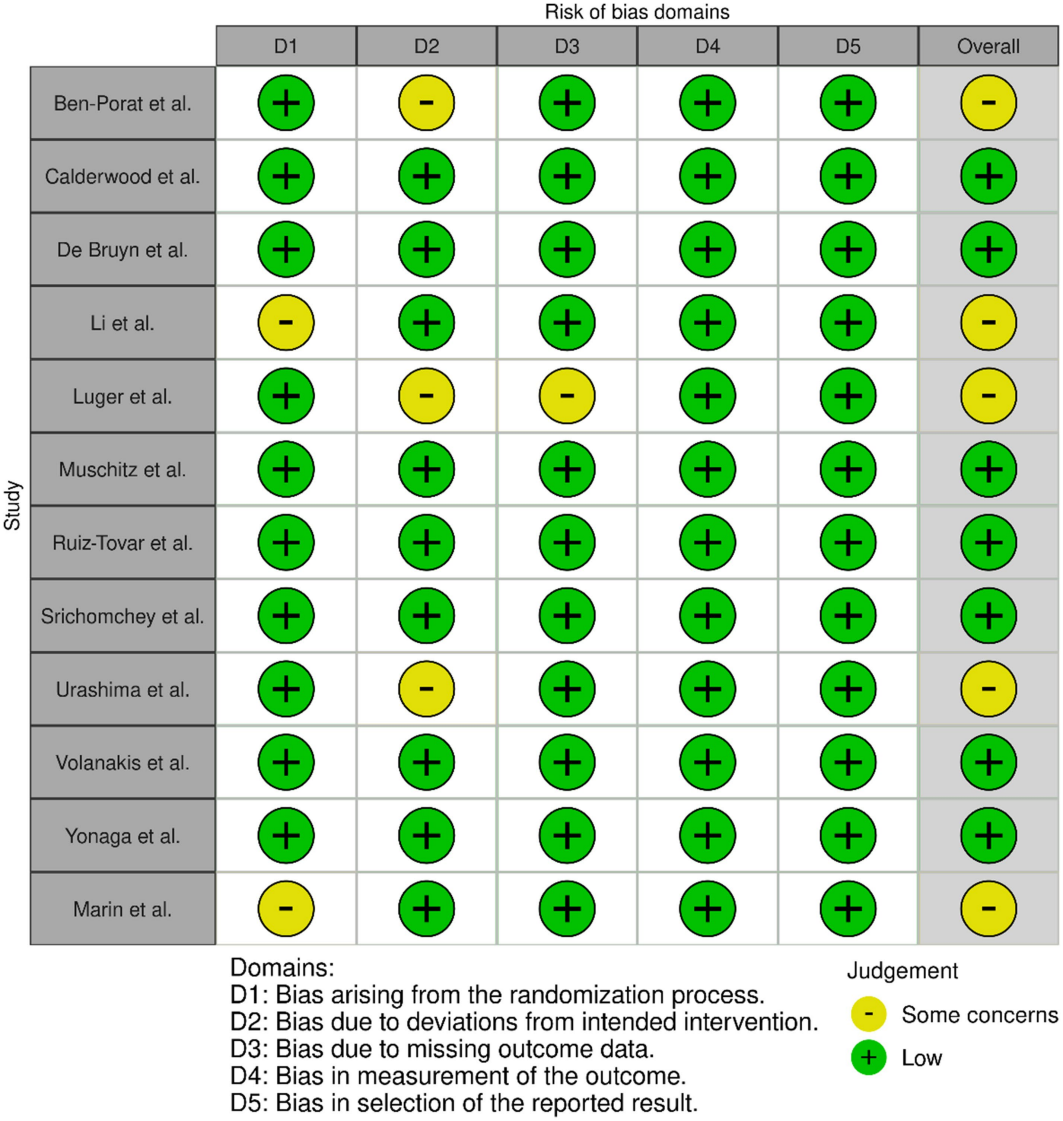
Traffic light plot summarizing risk of bias for the trial described in each article.

Risk of bias for each trial is summarized in Figure 2; 6 of the 11 trials were determined as low risk overall while 5 had some concerns. The trial of Ben-Porat et al. (45) used an open-label system meaning that both the researchers and the participants knew the treatment allocation. Additionally, a survey was done on the participants showing they had variable postoperative vitamin D intake from the diet and variable sun exposure which may have also affected the results. The trial of Li et al. (48) used a sealed envelope system of randomization which has been shown to be prone to deliberate tampering by researchers (57). In the trial of Luger et al. (49) there was a 19.6% dropout rate across both groups of participants and the reasons for these dropouts are not fully explored. Also, adherence to vitamin D supplementation decreased to 61% over the 6 months following surgery making the results less reliable. In the trial of Urashima et al. (54) 3.6% of patients in the intervention group and 5.4% in the placebo group stopped medication during the trial making it harder to properly compare them. The post-hoc analysis of the same trial by Yonaga et al. (55) did not have these same issues as it excluded patients who did not have specimens available for histopathological evaluation. The randomization process in the trial of Marin et al. (56) was not fully described.

## Discussion

As far as we are aware this is the first systematic review of perioperative micronutrients and post-surgery complications in patients undergoing gastrointestinal surgery. We anticipated studies of pre-, post- or perioperative oral or intravenous administration of single or mixed micronutrients reporting on short-term in-hospital complications such as infections and poor wound healing and on clinical outcomes such as duration of hospital stay. In a search limited to publications since 2014, one trial investigated preoperative administration (45), two investigated preoperative administration followed by prolonged post-discharge administration (50, 56), one investigated intravenous administration in the immediate postoperative period (48) and one used micronutrient containing dressings during the surgery itself (51). The other 6 trials (reported in 7 articles) began prolonged micronutrient administration some period after surgery/after discharge from hospital (46, 47, 49, 52–55). Among the 11 trials, only two looked at short-term (in-hospital) complications and outcomes (48, 51), one of these being the trial of micronutrient containing dressings (51). The other 9 trials investigated long-term (post-discharge) outcomes including bone health (45, 49, 50, 53), disease recurrence (46, 47) and survival (54, 55). Therefore, this systematic review is not able to identify whether perioperative micronutrients reduce the risk of in-hospital complications and/or improve in-hospital outcomes. Given that different micronutrients support immunity and wound healing and control adverse inflammation and oxidative stress, such benefits from micronutrients are possible, but the lack of trials on this indicates an important unanswered research question. Vitamin D was the most studied micronutrient with 8 trials (results reported in 9 articles) examining its use (45–47, 49, 50, 52–55). Multiple vitamins or micronutrients were used in 3 trials (45, 48, 56), one of these in combination with vitamin D (45). Five trials were conducted following bariatric surgery (45, 49, 50, 53, 56), five (6 articles) in patients undergoing surgery for gastrointestinal cancer (46, 48, 51, 52, 54, 55) and one in patients undergoing surgery for Crohn’s disease (47). Hence, the modest literature base identified (12 articles from 11 trials) is very heterogeneous in terms of micronutrients studied; types of patient included; route, duration and timing of initiation of administration; duration of follow-up; and outcomes reported. Additionally, some trials did not give the control group any “dummy” treatment (50, 52), others used a placebo (46–48, 54, 55) and others gave the control group lower doses of the micronutrient under study (45, 49, 53).

Vitamin D has a multitude of functions. It has an important role in maintaining the homeostasis of minerals as well as in bone mineralization as it stimulates absorption of phosphorus and calcium (58). Ben-Porat et al. (45) and Volonakis et al. (53) found that vitamin D supplementation did not affect the decrease in bone mineral density that can occur following bariatric surgery; the doses used in these trials were 4,000 IU/day for 2 months preoperatively (45) and 10,000 IU/day for the first postoperative month followed by 600 IU/day for a year (53). In contrast, Luger et al. (49) and Muschitz et al. (50), who both found that vitamin D improved bone health after bariatric surgery (as well improving emotional and physical wellbeing) used different dosing regimens. Luger et al. (49) gave three loading doses of 100,000 IU in the first month post-operation and then 3,420 IU/day for 5 months, while Muschitz et al. (50) gave 28,000 IU per week for 8 weeks pre-surgery and then 16,000 IU per week for 2 years post-surgery. Therefore, it is possible that vitamin D supplementation benefits bone health and quality of life following bariatric surgery but only if used at sufficiently high doses for sufficient duration. Additionally, the use of sublingual administration (as opposed to oral) by Muschitz et al. (50) may have allowed for an enhanced effect of vitamin D.

Vitamin D has also been shown to be protective in some forms of cancer (58) and to have anticancer effects such as decreasing tumor invasiveness and angiogenesis (59) as well as preventing proliferation and encouraging differentiation of cancer cells (60). However, Calderwood et al. (46) found that postoperative daily vitamin D for at least 4 months following endoscopic surgery did not decrease colorectal adenoma recurrence. It is worth noting this trial used a relatively low dosage (1,000 IU/day) compared to those that showed improvements in bone health. In contrast, Yonaga et al. (55) found that oral vitamin D (2,000 IU/day for 7 years) did prevent relapse and improve mortality in patients with poorly differentiated adenocarcinomas of the digestive tract. This may indicate that providing daily vitamin D after gastrointestinal cancer surgery can prevent cancer recurrence but specifically digestive tract cancer or poorly differentiated cancer. Additionally, Yonaga et al. (55) had a longer follow-up period (7 years post-surgery) compared with Calderwood et al. (3–5 years post-surgery) (46) so it may be the case that the use of Vitamin D is cancer protective but only over a prolonged timeframe.

Both the innate and adaptive immune responses have been shown to be influenced by vitamin D (25, 26). Cells involved in innate immunity such as neutrophils, macrophages and dendritic cells express vitamin D receptors and can also activate vitamin D allowing for control of the immune response (61). Vitamin D has also been shown to inhibit proliferation of adaptive immune cells such as T cells and to promote T regulatory (Treg) cells (62). Srichomchey et al. (52) found that postoperative vitamin D increased prevalence of Treg cells and promoted Treg associated cytokine IL-10 indicating that vitamin D may help modulate the immune response following surgery. In this context, de Bruyn et al. (47) found that postoperative vitamin D did not prevent recurrence of Crohn’s disease following ileocolonic resection. This is despite a high dose (25,000 IU/week) being used and is a surprising observation because vitamin D supplementation has been associated with increased anti-inflammatory peptide cathelicidin and decreased intestinal permeability both contributing to decreased inflammation in inflammatory bowel disease (63).

As previously mentioned, many micronutrients are capable of attenuating inflammation and oxidative stress (11). In accordance with this, Li et al. (48) found the use of intravenous multivitamins decreased levels of polar metabolites that indicate oxidative stress. In contrast, Marin et al. (56) found that a mix of multiple micronutrients did not reduce systemic inflammation. The exact micronutrients used, their doses and the duration of administration as well as type of patient and surgery might influence the impact on inflammation. Interestingly, Marin et al. (56) did find that micronutrient supplementation improved iron metabolism following surgery. The supplement used provided twice the RDA of the micronutrients provided, including iron, so increased iron intake may explain the improvement in iron metabolism. However, some of the other micronutrients included may also be relevant to this effect. For example, previous literature has established that vitamin A is involved in iron homeostasis and vitamin A deficiency reduces renal erythropoietin thereby inhibiting erythropoiesis (64). Therefore, the vitamin A present in the mix used by Marin et al. (56) may contribute to the observed effect on iron homeostasis following surgery.

Vitamin E has antioxidant and anti-inflammatory properties (65). One mechanism of vitamin E action is the inhibition of the cyclooxygenase-2 enzyme which is a promotor of inflammation. Vitamin E may also help prevent infection as high-dose administration has shown to increase levels of anti-inflammatory CD47 and allow for shorter hospitalization times in patients with pneumonia (66). Additionally, vitamin E has been shown to reduce pain and infection in wounds caused by burns (67). Silicone has also shown to have anti-inflammatory and antioxidant properties (68). Ruiz-Tovar et al. (51) found that the use of the combination of vitamin E and silicone in wound dressings helped reduce postoperative pain, infection and inflammation. More research could be done on different routes of vitamin E and silicone administration, such as oral, sublingual and intravenous, and investigating whether this combination could reduce general postoperative infection and pain rather than specifically within wounds.

This systematic review was restricted to RCTs which are able to demonstrate a causal effect (e.g., between micronutrient administration and post-surgery outcome). Thus, this is a strength. However, the systematic review also has some limitations. One of these was the use of only two databases to identify relevant literature; nevertheless, those databases identified a significant literature base to select relevant articles from (*n* = 2,750 articles after de-duplication). Furthermore, the search was limited to articles published after January 1st 2014. This was done in order to focus on only the most up to date literature that would be relevant to current clinical practice (pre-surgical preparation, surgical interventions, post-surgery care, nutrition support etc.); nevertheless, some relevant articles from prior to 2014 will have been missed. Finally, only articles written in English were included; any relevant articles published in other languages will have been missed.

The research included in the systematic review was fairly restricted in the micronutrients studied, with significant emphasis on vitamin D. Future research could incorporate other micronutrients such as vitamins C and A. Vitamin C has been shown to help enforce epithelial barriers against the invasion of pathogens, to protect against oxidative stress and to promote wound healing (35) so it could be useful in preventing surgical infections. Other micronutrients important for immune support include zinc, copper and selenium (27–30, 33, 34) and these have not been well studied in the context of post-surgical complications. Vitamin A is known to be anti-inflammatory (69) making it a potential candidate for management for postoperative inflammation. The vast majority of the included trials used oral administration; it would be interesting to investigate other routes of administration. In particular, intravenous administration allows for 100% bioavailability by bypassing the body’s processes of digestion and absorption (70) which will be impaired following gastrointestinal surgery; thus the intravenous route may allow micronutrients to act more effectively.

The ability to draw firm conclusions from this systematic review is influenced by the limitations of the included trials. For example, the lack of trials of micronutrients during the pre- and immediate post-operative period and the lack of trials reporting short-term, in hospital complications has already been mentioned. Furthermore, there are few trials of micronutrients investigating immune function, inflammation and oxidative stress in this population. Some of the smaller trials may have been underpowered. In addition, all trials provided micronutrients at the same dose to their participants irrespective of age, sex, body size and disease state, factors which might affect micronutrient requirements and metabolism, although this is poorly described. Although requirements (as μg or mg/day) for some micronutrients (e.g., vitamins B12, C and folate) are stated to not differ between men and women, women have a higher requirement for iron but lower requirements for vitamins A and B6 and for zinc and selenium (71). None of the included studies tailored micronutrient dosage according to participant characteristics.

One area not addressed in any of the included trials is the gut microbiome. The gut microbiome interacts with the host’s immune and inflammatory systems (72–75) and is influenced by diet (76–78). The gut microbiome is altered in those living with obesity (79), colorectal cancer (80) and inflammatory bowel disease (81) and is altered following bariatric surgery (82, 83) and likely by other gastrointestinal surgeries. Gut microbes also need a supply of micronutrients, some micronutrients have been shown to influence the gut microbiome (84–91) and it is possible that perioperative micronutrients could affect host immunity and inflammation via changing the microbiome and this, in turn, could reduce post-surgery complications. Certainly, there is evidence for microbiome-targeted interventions such as probiotics and synbiotics to improve outcomes following gastrointestinal surgery (92, 93). Whether micronutrients can act by this mechanism to improve outcome in surgical patients should be explored.

## Conclusion

The prevention of surgical complications is economically valuable, improves the clinical experience for the patient and improves their quality of life following surgery. Micronutrients have been shown to have immune supporting and antioxidant and anti-inflammatory properties and deficiencies are associated with poorer clinical outcomes; therefore perioperative micronutrient provision is a possible strategy to help address surgical complications. This systematic review has highlighted possible applications of this approach, suggesting the potential for high dose vitamin D to prevent long-term bone loss following bariatric surgery and to prevent relapse of poorly differentiated carcinomas, although inconsistent findings and a low number of trials limits a conclusive statement around these effects; the use of multivitamins to reduce postoperative oxidative stress; and the use of vitamin E and silicone in wound dressings to reduce infection, pain and inflammation. That being said, there was a limited amount of research found and relevant trials differed greatly in design and outcome. More research is needed, perhaps with larger numbers of patients and focussing on a larger range of micronutrients and routes of administration to properly establish perioperative micronutrient supplementation as a way to improve outcomes in the period immediately following gastrointestinal surgery.

## Data Availability

The original contributions presented in the study are included in the article/supplementary material, further inquiries can be directed to the corresponding author.
